# Monitoring commitments for adolescent well-being: bridging the gaps between promise, implementation and impact

**DOI:** 10.1093/heapol/czag007

**Published:** 2026-09-30

**Authors:** Thahira Shireen Mustafa, Gareth Jones, Lucy Fagan, Philip Idoko, Petar Mladenov, Arlene B Tickner, Kathleen Sherwin, Anshu Mohan, Rajat Khosla

**Affiliations:** The Partnership for Maternal Newborn & Child Health (PMNCH), World Health Organization, Avenue Appia 20, CH 1211 Geneva 27, Switzerland; The Partnership for Maternal Newborn & Child Health (PMNCH), World Health Organization, Avenue Appia 20, CH 1211 Geneva 27, Switzerland; School of Medicine, Newcastle University, Framlington Place, Newcastle upon Tyne NE2 4HH, United Kingdom; Major Group for Children and Youth, United Nations Plaza, New York 10017, United States; Royal Free London NHS Foundation Trust, Pond Street, Rosslyn Hill, London NW3 2QG, United Kingdom; Education as a Vaccine (EVA), Plot 1534, Cadastral Zone B8, Jahi District, Katampe, Abuja, Nigeria; Ministry of Youth and Sports, Government of Bulgaria, 75 Vasil Levski Boulevard, 1142 Sofia, Bugaria; Ministry of Foreign Affairs, Government of Colombia, Palacio de San Carlos, Calle 10 #5-51 Bogotá D.C., Colombia; The Partnership for Maternal Newborn & Child Health (PMNCH), World Health Organization, Avenue Appia 20, CH 1211 Geneva 27, Switzerland; Chief Strategy & Engagement Officer, Plan International, Discovery House, 28-42 Banner Street, London, EC1Y 8QE, United Kingdom; The Partnership for Maternal Newborn & Child Health (PMNCH), World Health Organization, Avenue Appia 20, CH 1211 Geneva 27, Switzerland; The Partnership for Maternal Newborn & Child Health (PMNCH), World Health Organization, Avenue Appia 20, CH 1211 Geneva 27, Switzerland

**Keywords:** adolescent health, youth, well-being, commitment, accountability, policy

## Abstract

This paper presents a timely and critical analysis of the translation of global commitments made at the landmark 2023 Global Forum for Adolescents into national action to prioritize adolescent well-being. The Partnership for Maternal, Newborn & Child Health is monitoring implementation of these commitments. Employing a mixed methods approach, the paper examines the nature of these multi-stakeholder commitments and their early implementation in four diverse country contexts, specifically Bulgaria, Colombia, Malawi, and Nigeria. Key findings highlight the critical role of multi-sectoral coordination, the imperative of fostering genuine adolescent and youth engagement, and the complexities inherent in managing policy synergies and trade-offs within varied national realities. The analysis reveals that while the Global Forum for Adolescents successfully catalyzed unprecedented attention and pledges for adolescents, effective and sustainable realization of these commitments at the national and local level is an urgent imperative contingent upon overcoming persistent structural barriers and adopting adaptive, systems-informed policy responses. The paper examines the roles of various actors including governments, civil society, and young people, and concludes with a set of recommendations aimed at supporting systemic, long-term change through enhanced cross-sectoral collaboration and integrated policies and programs; and underscores the importance of a continued focus on accountability for adolescent well-being.

Key messages

- Adolescents are a unique, underserved demographic. The 2023 Global Forum for Adolescents secured vital commitments from national governments and others for actions to meet the well-being needs of adolescents. This article provides an early assessment of actions taken related to the commitments made by four countries.
- Pledges face persistent structural barriers. The country case studies reveal common challenges: fragmented systems, funding gaps, and poor data.
- Meaningful adolescent and youth engagement and multi-sectoral action are essential for impactful, equitable outcomes.
- Accountability requires adaptive governance and robust data. Strong monitoring and evaluation, disaggregated data, and stable leadership are non-negotiable for sustained progress.

## Introduction: the unprecedented focus on adolescent well-being

Today's 1.3 billion adolescents (ages 10–19) represent the largest cohort in history, yet their needs remain under-recognized in global agendas (Baird et al. 2025). Neither the Millennium Development Goals (MDGs) nor the Sustainable Development Goals (SDGs), gave adolescents explicit priority, and limited disaggregated data has reinforced their invisibility (Guglielmi and Jones 2019, Guglielmi et al. 2021). This exclusion is particularly critical as we are now only four years away from the 2030 expiration of the SDGs, many of which are currently at risk of non-attainment. Historically, this oversight stemmed from a tendency to either group adolescents with younger children (e.g. 0- to 14-year-olds) or with broader “youth” (15–24 years) or “adult” (15 years+) categories, blurring the unique biological, cognitive, and social transformations that define ages 10–19. However, this period of adolescence is characterized by distinct vulnerabilities, particularly concerning sexual and reproductive health risks, mental health conditions, and exposure to violence, as well as critical opportunities to establish lifelong healthy behaviors (Patton et al. 2018). Addressing these requires targeted interventions distinct from those for children or adults.

A more central positioning of the adolescent agenda has recently emerged, largely driven by the advocacy and mobilization efforts of the Partnership for Maternal, Newborn & Child Health (PMNCH) and its partners. The 2023 Global Forum for Adolescents (GFA) was a pivotal moment, correcting the long-standing neglect of adolescent well-being in global health policy (PMNCH 2023c, 2023d). Unlike earlier agendas that subsumed adolescents under broader themes such as nutrition or sexual and reproductive health and rights (SRHR), the GFA explicitly prioritized their comprehensive needs, underscoring recognition of the demographic dividend's potential through targeted investment (Dyke et al. 2021). The primary aim of the GFA was to catalyze high-level political and financial commitments and foster global accountability for the world's 1.8 billion young people. As part of the “1.8 Billion Young People for Change Campaign,” it mobilized diverse partners, engaged over one million young people, and generated commitments from governments, civil society, and private actors alongside grassroots demand, creating momentum for sustained accountability (PMNCH 2022, 2023a, 2023b, 2023c, 2023d, Saran et al. 2026). Crucially, the GFA amplified the adolescent well-being framework (Ross et al. 2020) and related knowledge products, which provide a holistic conceptual foundation for these efforts. This framework moves beyond a narrow health focus to encompass five interconnected domains: (i) good health and optimum nutrition; (ii) connectedness, positive values, and contribution to society; (iii) safety and a supportive environment; (iv) learning, competence, education, and skills; and (v) agency and resilience. By fostering broad awareness and demand for action, the GFA enabled the effective integration of adolescent well-being into the current SDG discourse and is beginning to shape the nascent post-2030 global development architecture.

Two years on, the urgency is to determine whether these pledges are translating into national action. PMNCH is monitoring early implementation to ensure that the momentum catalyzed in 2023 is sustained and strengthened. The purpose of this study is to evaluate the early-stage implementation progress of the national commitments made at the GFA, identifying the political, institutional, and resource-related factors that facilitate or hinder the translation of global pledges into meaningful national-level change.

## Methods

This paper utilizes a multi-method qualitative approach, incorporating descriptive quantitative mapping to investigate to thoroughly investigate early progress pertaining to the commitments related to adolescent well-being that were made at the GFA. While the analysis is primarily qualitative, it follows a mixed-methods logic by integrating a macro-level descriptive analysis of commitments across 24 countries with in-depth qualitative case studies of four selected countries. This breadth-and-depth approach provides a systematic content analysis combining the macro-level landscape of commitments with an in-depth examination of specific national contexts, by assessing the micro-level realities of their implementation within four selected countries. This was done to understand the thematic and structural alignment of the commitments against the seven priorities of the youth-defined “Agenda for Action for Adolescents” launched during the GFA. This analysis allowed for the quantitative mapping of commitment priorities and an understanding of the comprehensive multi-sectoral nature of the commitments.

### Macro-level content analysis and quantitative mapping

The first phase involved a systematic content analysis of all public-facing GFA commitments from the 24-country cohort (*N* = 24). The analytical procedure involved coding each country's pledge against the seven priorities of the youth-defined “Agenda for Action for Adolescents,” which spans health services, education, agency, and social protection. This enabled a descriptive quantitative mapping of commitment priorities, identifying the frequency and distribution across specific sectors (e.g. the prevalence of health-focused versus social protection-focused pledges). The results of this quantitative mapping are presented in Section *‘Nature and scope of commitments: political, financial, and programmatic pledges’* to provide a structural baseline of the GFA commitment landscape.

### Case study selection and qualitative data collection

The qualitative “deep dive” data focused on four countries (Bulgaria, Colombia, Malawi, and Nigeria). These were selected using a combination of stratified purposive sampling, to ensure a wide geographical perspective (Africa, Europe, and Latin America); and maximum variation sampling, to capture progress against a wide range of commitment types across multiple domains of the adolescent well-being framework, implementation approaches, and observed outcomes. In-depth qualitative case studies of a limited number of countries aimed to enable the identification of common themes, patterns, and causal mechanisms that may be applicable to other contexts sharing similar characteristics.

For each case study, we conducted a systematic review of national policies, strategic plans, legislative documents, progress reports, program evaluations, and other official records to gather in-depth qualitative information on progress since the commitments were made. These findings were triangulated against reported realities from key informant (KIs) interviews to identify potential discrepancies between stated policy intentions and implementation progress.

### Key informant interviews and data validation

The qualitative insights were primarily derived from structured phone interviews with a limited number of key informants (KI), including commitment makers (high-level government officials or institutional leaders), technical experts, and youth network representatives from the respective case study countries. These KIs were specifically contacted for fact-checking and to provide updates and nuanced insight regarding progress against their national commitments.

A crucial element of the validation process was the involvement of selected KIs from Bulgaria, Colombia, and Nigeria who contributed as co-authors. These co-authors provided a layer of internal validation, verifying that the contextual statements and analysis regarding the situation in their respective countries were accurate and reflected the on-the-ground realities. For Malawi, information and insights were leveraged from the comprehensive PMNCH-supported Collaborative Advocacy Action Plan (CAAP) initiative process. This involved a recent review of national commitments and synthesis of feedback from extensive consultative workshops with a diverse array of national organizations working on women's, children's, and adolescents’ health, government representatives, and community groups, including youth, persons with disabilities, media, and civil society organizations.

This process enabled the case studies to go beyond uncritical repetition of policy and program reports to uncover the underlying narratives of the why and how of progress or lack thereof, including the influence of political will, community engagement, resource allocation dynamics, and unforeseen barriers or facilitators.

## Results

### Nature and scope of commitments: political, financial, and programmatic pledges

The commitments made at the GFA reflect a multi-sectoral mobilization across 24 national governments and various non-state actors. Analysis of the 24-country cohort (*N* = 24) identified distinct thematic pledges mapped against the seven asks of the youth-defined “Agenda for Action for Adolescents” (see Section *‘Alignment with the adolescent well-being framework and agenda for action for adolescents’*).

Pledges were secured from three primary categories of actors:

- **Governments:** 24 national governments made explicit commitments: Botswana, Bulgaria, Canada, Chile, Colombia, Ecuador, Ethiopia, Ghana, Honduras, Ireland, Lesotho, Liberia, Malawi, Mexico, Namibia, Nigeria, Portugal, Congo, Serbia, Sint Maarten (autonomous country within the Kingdom of the Netherlands), South Africa, United Republic of Tanzania, USA, and Zambia. These commitments often included specific policy changes, legislative actions, and dedicated budgetary allocations. On average, each country committed to four out of the seven priority asks. Analysis of these pledges reveals varying levels of prioritization across the framework. Ask 2 (health services) was the most frequent priority, addressed by 83.3% (*n* = 20) of the countries. This was followed by Ask 7 (strengthening agency) at 66.7% (*n* = 16), and Ask 4 (legal and policy reform) at 58.3% (*n* = 14). Conversely, Ask 5 (social protection) was the least addressed area, included in only 29.2% (*n* = 7) of national pledges. In terms of comprehensiveness, only 20.8% (*n* = 5) of countries (Colombia, Ghana, Lesotho, Malawi and Tanzania) submitted “all-encompassing” pledges covering six out of the seven priority asks.
- **Regional and inter-governmental bodies:** Three regional bodies; the African Union, the European Commission, and the African Leaders Malaria Alliance also made commitments, emphasizing integrated planning and regional coordination for adolescent well-being.
- **Non-state actors:** This broad category encompassed:
  - **Private sector/philanthropic organizations:** This included Born This Way Foundation, Cotton On Foundation, FIA Foundation, Fondation Botnar, Goleadoras Foundation, Mariwala Health Initiative, MTV Entertainment Studios, MTV Staying Alive (supported by The Bill & Melinda Gates Foundation), One Earth, Prospira Global, Surgo Health, Pivotal Ventures, The Wellbeing Foundation Africa in partnership with Reckitt, and UNICEF & Generation Unlimited. Their pledges often focused on specific areas such as safe cities, digital environments, mental health, road safety, climate action, and sexual and reproductive health.
  - **Non-governmental organizations:** The Christian Council of Nigeria, Plan International, and Women Deliver committed to advancing adolescent well-being, often with a focus on girls' rights, education, protection, and community-based initiatives.
  - **Healthcare professional associations:** The International Pediatric Association made commitments to improving adolescent well-being with a particular emphasis on health, focusing on service provision and quality.

These pledges encompassed a range of commitment modalities, including direct financial investments, legislative and policy changes, and specific programmatic actions (PMNCH 2023c, 2023d).

### Alignment with the adolescent well-being framework and agenda for action for adolescents

The GFA commitments were notable for aligning closely with the five domains of the adolescent well-being framework: good health and optimum nutrition; connectedness, positive values and contribution to society; safety and a supportive environment; learning, competence, education, skills and employability; and agency and resilience (Ross et al. 2020).

The commitments also responded directly to the responses to the *What Young People Want* Survey (Saran et al. 2026) and the youth-defined “*Agenda for Action for Adolescents*” (PMNCH 2023a, 2023b, 2023c, 2023d), reinforcing the Forum's emphasis on adolescent co-leadership. Table 1 serves as a detailed matrix of these commitment types, categorizing national pledges against the seven “asks” of the youth-defined “*Agenda for Action for Adolescents*.” By mapping each country's thematic focus of the commitments ranging from vocational training to social protection, this table provides a granular overview of the specific policy and programmatic priorities for each nation. Collectively, the 24 countries addressed all seven “asks”, spanning vocational training, quality health services, mental well-being, legal reform, social protection, stigma reduction, and youth agency.

**Table 1:** Summary of commitments against the seven asks of the agenda for action for adolescents.

Country	Commitments matrix against the asks of the agenda for action for adolescents^a,b^						
	Ask 1—Providing vocational training and secondary/third-level education	Ask 2—Providing affordable, high-quality adolescent health and well-being services	Ask 3—Ensuring mental well-being and resilience	Ask 4—Reforming harmful and punitive laws and institute affirmative and protective legal and policy mechanisms	Ask 5—Strengthening and establishing social protection mechanisms	Ask 6—Preventing stigma and discrimination	Ask 7—Strengthening agency of adolescents and youth
Botswana	✓	✓	✓	✓			
Bulgaria		✓	✓		✓		
Canada		✓		✓		✓	✓
Chile		✓	✓			✓	✓
Colombia		✓	✓	✓	✓	✓	✓
Ecuador		✓		✓		✓	
Ethiopia				✓			
Ghana	✓	✓		✓	✓	✓	✓
Honduras	✓					✓	
Ireland		✓	✓			✓	✓
Lesotho		✓	✓	✓	✓	✓	✓
Liberia	✓	✓		✓	✓	✓	
Malawi	✓	✓		✓	✓	✓	✓
Mexico	✓	✓				✓	✓
Namibia		✓					
Nigeria	✓	✓					✓
Portugal	✓		✓				
Republic of the Congo	✓	✓	✓			✓	✓
Serbia		✓		✓			✓
Sint Maarten		✓	✓				✓
South Africa	✓			✓		✓	✓
Tanzania		✓	✓	✓	✓	✓	✓
USA	✓	✓		✓		✓	✓
Zambia	✓	✓		✓		✓	✓

^a^The checkmark (✓) indicates that the national government made an explicit political, financial, and/or programmatic commitment addressing this specific priority area aligned with the 2023 GFA.

^b^The asks indicate the priorities representing the youth-defined *Agenda for Action for Adolescents*, developed through the *What Young People Want* initiative, which mobilized over 1.2 million young people globally, and launched during the GFA.

### Global to local: early progress, challenges, and lessons learned

Translating national commitments into tangible action is complex, with diverse approaches and ongoing challenges. To evaluate how these macro-level pledges translate into national action, we conducted in-depth case studies of Bulgaria, Colombia, Malawi, and Nigeria. As detailed in Methods (Section *‘Key informant interviews and data validation’*), the evidence for these cases was derived from a systematic review of post-2023 national policies, strategic plans, and legislative records, triangulated with structured interviews from KIs who contributed to this study as co-authors or expert validators.

While these nations have taken significant steps to advance adolescent well-being, achieving comprehensive outcomes remains a work in progress (Table 2). These examples offer insights into early progress, implementation strategies, remaining barriers, and the factors that facilitate or hinder effective implementation.

**Table 2:** Summary of implementation efforts and early progress in case study countries.

Country	Timeline	Early progress against commitments
Bulgaria	2021–30	Progress is centered on the National Youth Strategy, a 10-year policy horizon. Implementation is evidenced by the scaling of the UPSHIFT innovation program, which has reached a significant milestone in regional expansion during the 2023–24 cycle.
Colombia	2023–26	Implementation is driven by a mid-term statutory reform horizon, specifically Law 2328 of 2023. This law codifies adolescent health rights into the national legal framework, with current efforts focusing on the 2024–25 regulatory rollout.
Malawi	2024–27	Evidence shows a focus on a 3-year advocacy and budgetary window. The CAAP serves as the primary mechanism for monitoring financial commitments to RMNCAH, with the first fiscal review occurring in late 2024.
Nigeria	2019–24	Progress is marked by the finalization of the current policy cycle, including the enactment of the revised National School Health Policy. The institutionalization of annual Intergenerational Dialogues provides a recurring timeline for youth-led accountability through 2024 and beyond.

#### Case study: Bulgaria—integrated programs for youth engagement

Bulgaria's commitments span multiple domains of the adolescent well-being framework, including improving access to affordable health services, strengthening social protection, promoting mental resilience, and expanding adolescent and youth participation (Republic of Bulgaria 2024). The government also pledged to foster inclusive youth engagement, build a national evidence base, and institutionalize whole-of-government approaches for measurable outcomes.

These pledges build on Bulgaria's history as an advocate for child and youth rights, both internationally through leadership roles at the UN; and domestically, via mechanisms such as the inter-institutional working group on the UN Convention on the Rights of the Child (UNICEF 2025a).

The National Youth Strategy 2021–30 provides the overarching framework, coordinated by the Ministry of Youth and Sports, and was developed following extensive youth consultations and public review (Republic of Bulgaria 2021, National Assembly of the Republic of Bulgaria 2023). Recent reforms have broadened funding eligibility to municipalities and informal youth groups, helping decentralize youth empowerment beyond urban centers (European Commission 2025a). Investments in International Youth Centers, co-financed through the Recovery and Resilience Plan, have expanded youth infrastructure, with eight centers already operational and 20 more planned (Republic of Bulgaria 2024). These hubs provide training, health support, and international exchange opportunities. UNICEF's UPSHIFT program, launched in 2024–25, further supports adolescent problem-solving and entrepreneurship, reflecting a growing emphasis on agency and participatory design (UNICEF 2025a).

Yet challenges remain. Social protection and welfare policies are still siloed, undermining cross-sectoral responses (The World Bank 2019, OECD 2022). While Bulgarian youth centers reach large numbers, with over 50 000 young people attending them in 2023, their long-term impact remains contested (Mahoney et al. 2001, Villa 2024). Political instability and successive interim governments have disrupted policy continuity and weakened trust among young people, only 21% of whom report interest in formal politics (Kobeszko 2024, Popivanov et al. 2024). Monitoring systems also lag, with biennial reports too slow to enable timely adjustments, though a new feedback-oriented platform is planned for 2026 (European Commission 2025b).

Overall, Bulgaria demonstrates clear normative leadership and growing prioritization of youth participation and policy frameworks. However, a recent youth work development report by the European Youth Forum (2025) and statement by the Deputy Prime Minister Petrova (2025) suggest that persistent fragmentation, governance volatility, and weak accountability mechanisms continue to limit the translation of strong commitments into sustained improvements in adolescent well-being.

#### Case study: Colombia—legislative action

The Government of Colombia pledged to involve adolescents in political decision-making through participatory budgeting and accountability, to guarantee resources for its State Policy for Comprehensive Development, and to implement the *Jóvenes En Paz* program for vulnerable youth, aimed at addressing inequalities and promoting peace in marginalized regions (Gobierno de Colombia 2024).

Colombia's agenda is underpinned by robust legislative action. Law 2328 (2023) guarantees funding for adolescent development and prioritizes youth needs across government sectors, supported by Resolution 051 (2023) and Circular 044 (2022), which institutionalize adolescent-responsive health services, particularly SRHR (ICBF 2023a, 2023b). These reforms expanded access to contraception and strengthened youth-friendly service models. A 2022 Constitutional Court ruling decriminalizing abortion up to 24 weeks further reduced stigma and expanded adolescent reproductive rights (Centre for Reproductive Rights 2022).

Institutionally, Colombia created the Presidential Advisory for Youth and a Vice-Ministry of Youth within the new Ministry of Equality and Equity, consolidating a rights-based, intersectional approach that addresses gender, race, ethnicity, disability, and geography (Gobierno de Colombia 2024). These structures advance youth participation, coordinate intersectoral work, and ensure accountability.

A defining feature of Colombia's approach is participatory policymaking. Young people, especially from marginalized groups, have co-designed mental health and SRHR services, including innovations in La Guajira, a department in the Northeast Region of the country (Agudelo-Hernández et al. 2025). A donor coordination platform also gives youth equal footing in discussions on international cooperation (PoD Consortium 2025). The *Jóvenes En Paz* program, targeting 14- to 28-year-olds in high-risk contexts, aims to guarantee universal access to health, education, and training while promoting disarmament and peaceful coexistence, offering a tangible example of resources allocated to equity and non-discrimination (Gobierno de Colombia 2025).

Despite these advances, structural challenges persist. Inequality, poverty, and education gaps disproportionately affect rural, Afrodescendent, and indigenous adolescents, contributing to dropout, early pregnancy, and child labor (DANE 2022a, 2022b, 2025, UNFPA Colombia 2024). Armed conflict and displacement exacerbate mental health issues, child recruitment, and gender-based violence (Praveen 2022, Weber et al. 2025). Rising adolescent mental health problems further reflect the psychosocial toll of violence and instability (Gómez-Restrepo et al. 2023, Martínez et al. 2024).

These realities highlight the limits of legislative progress without multi-sectoral integration and strong local implementation. To close the gap, the government is investing in a registry and monitoring system for conflict- and gender-based violence (ICMEC 2022), strengthening evidence-informed policy, and embedding youth-led accountability mechanisms (Padilla et al. 2022, DANE 2022a, 2022b).

Colombia also signaled its global leadership by hosting the first Global Ministerial Conference on Ending Violence Against Children in 2024, further amplifying youth voices and advancing international accountability (Government of Colombia 2024).

Colombia illustrates the potential of a legislative and participatory governance model for adolescent well-being but also demonstrates that in contexts marked by inequality and violence, coherence across sectors, sustained financing, and meaningful youth engagement are essential to move from commitments to impact.

#### Case Study: Malawi—budgetary allocation

Malawi illustrates how budgetary commitments both enable and test progress on adolescent well-being within a constrained health system. In 2023, the Government pledged major increases in funding for gender, social protection, nutrition, SRHR, and family planning, alongside policies to address menstrual health, child marriage, adolescent pregnancy, mental health, and financial inclusion (Government of Malawi 2023). Other priorities included expanding internet connectivity, strengthening education retention, building climate resilience, and institutionalizing youth participation through youth parliaments.

Fiscal alignment with global priorities is improving. While Malawi has consistently fallen short of the Abuja target of allocating 15% of its annual budget to health (UNICEF Malawi 2023a), SRHR funding has grown, supported by the 2023–30 Health Financing Strategy (Republic of Malawi 2023, 2024). Health sector spending rose to 8.7% of the national budget in 2023/24 and 9.2% in 2024/25 (UNICEF Malawi 2024b). Social protection allocations more than doubled between 2022/23 and 2023/24, with expanded coverage of the Social Cash Transfer scheme, though targets remain unmet. By contrast, nutrition remains underfunded at only 0.6% of the national budget (UNICEF Malawi 2024a, 2024b). These trends signal recognition of the importance of resource allocation but highlight uneven progress across sectors.

Financial investments, however, have not translated into clear gains in adolescent outcomes (UNICEF 2025b). Structural weaknesses persist: chronic underfunding, shortages in health workers, and inequitable rural access constrain delivery (Amref Health Africa and PMNCH 2024). While taxes on sanitary products were removed in 2022, levies on raw materials still impede local production, keeping costs high (UNFPA 2025).

Adolescent girls remain especially vulnerable. Maternal, neonatal, and child mortality remain high, with adolescent pregnancy a leading driver of school dropout (29% of girls aged 15–19 reported early pregnancies in 2024) (UNICEF 2025b). Socioeconomic challenges compound these risks: youth face limited employment opportunities, persistent early marriage, and barriers to education and healthcare (Chunga and Evance 2025). Implementation of vocational training policies (TEVET) is hampered by limited technical and financial capacity (Mpinganjira 2025).

In response, Malawi has emphasized multi-sectoral coordination. The CAAP, led by the Ministry of Health with civil society, provides a platform for cross-sector accountability and youth engagement (Amref Health Africa and PMNCH 2025). The CAAP supports co-design and monitoring of policies by adolescents, communities, and frontline workers. This reflects recognition that adolescent well-being requires integrated action across health, education, nutrition, and economic empowerment. Yet coordination bottlenecks persist: ministries often operate in silos, and institutionalization of cross-sector collaboration is incomplete (UNICEF Malawi 2023a, 2023b).

Malawi's case highlights a paradox. Political and financial commitments are substantial, but without resilient systems, strong coordination, and effective implementation, progress on adolescent health, education, and empowerment will remain uneven. The experience underscores that sustainable gains depend not only on increased budgets but also on integration, accountability, and adaptive systems.

#### Case study: Nigeria—youth-led accountability

Nigeria has signaled strong national intent to prioritize adolescent well-being through explicit policy commitments and increased youth participation in governance processes. In its 2023 pledge during the GFA, the government committed to mainstream adolescent priorities into national development strategies, strengthen disaggregated data systems, and institutionalize youth engagement in the design, implementation, and monitoring of adolescent-focused policies (Federal Ministry of Health Nigeria 2023). These commitments align with the National Youth Policy (2019–23), which emphasized a multi-sectoral, rights-based approach to youth development and advocated for integrated service delivery, particularly across health, education, protection, and employment sectors (Federal Ministry of Youth and Sports Development Nigeria 2019).

Nigeria's CAAP, co-convened by the Federal Ministry of Health and national and international partners, facilitates structured dialogue between young people, civil society, and policymakers to ensure youth-led accountability. Through the CAAP, youth advocates have secured timely family planning funds and pushed for a dedicated adolescent well-being budget line (AHBN and PMNCH 2025). They have contributed to strategic national documents, advocated for the review of the National Policy on Adolescent Health (2021–25), and strengthened advocacy skills among peers, increasing visibility of adolescent health issues through social and traditional media.

Youth-led initiatives have also shaped Nigeria's HIV response. The #UPROOT scorecard, developed by the Association of Positive Youth in Nigeria, assesses national HIV programs for young people, providing actionable data for the Global Fund Country Coordinating Mechanism (The Youth Pact 2023). Peer-led organizations, including the Association of Adolescent and Young Persons Development, facilitate youth-friendly HIV testing and access to prevention services such as condoms and pre-exposure prophylaxis. These initiatives have strengthened collaboration with partners, enhanced legitimacy of youth-led data, and raised awareness of protective laws and policies through targeted media campaigns (The Youth Pact 2023, UNAIDS 2024). With youth (15–24 years) representing approximately 24% of new HIV infections in 2023 (UNAIDS 2025), youth engagement is critical for responsive programming.

Beyond national policy spaces, youth-led accountability extends to humanitarian settings, particularly in Nigeria's conflict-affected North-East. Under the Accountability to Affected People framework, youth associations participate actively in decision-making, leading reforms such as multilingual 24/7 reporting hotlines, trained community focal points, and inclusive consultations prioritizing girls and young people with disabilities (OCHA 2025). These reforms signal a shift from viewing youth as passive beneficiaries to recognizing them as essential actors in equitable programming.

Despite these advances, systemic barriers persist. Nigeria's reliance on donor funding for adolescent sexual and reproductive health, underinvestment from domestic sources, and uneven state-level capacity create financial and implementation volatility (AHBN and PMNCH 2024). Coordination across sectors and government tiers remains fragmented, limiting holistic responses to interlinked drivers of adolescent vulnerability, including poverty, gender-based violence, low school retention, and inadequate mental health services.

Nigeria's experience illustrates that sustainable adolescent well-being outcomes depend not only on policy intent but also on institutional reforms, predictable financing, multi-sectoral collaboration, and multi-level systems integration. Meaningful youth engagement embedded in formal accountability structures can drive political traction, enhance budget transparency, and pressure institutions to deliver on commitments, but long-term monitoring remains essential.

## Discussion

This study provides a critical and timely analysis of the early-stage implementation of commitments made at the 2023 GFA, a pivotal moment in global health policy for its explicit prioritization of young people’s unique needs. This diversity of commitment types reflects the fact that adolescent well-being requires a comprehensive, multi-sectoral approach that extends beyond financial allocations to encompass policy, legislative and program reforms, institutional strengthening, and direct service delivery improvements. The inherent multi-faceted nature of these commitments necessitates strong cross-sectoral coordination to ensure effective implementation. By examining the transition from global rhetoric to national reality, our findings reveal how the explicit prioritization of young people’s unique needs is beginning to reshape national policy architectures.

### Alignment with global frameworks

The structural alignment of national pledges with the adolescent well-being framework and the youth-defined “Agenda for Action” (as detailed in Section *‘Results’*) offers a common conceptual language for multi-sectoral planning. Our analysis suggests that this coherence is a significant maturation of global health policy; it moves beyond fragmented interventions to a holistic understanding of adolescent needs. However, the quantitative mapping in Section *‘Nature and scope of commitments: political, financial, and programmatic pledges’* reveals a “prioritization gap”. While 83% of countries focused on health services, only 29% addressed social protection. This interpretation suggests that while the Framework provides a holistic roadmap, national implementation remains heavily skewed toward traditional health-centric models, potentially leaving the socioeconomic determinants of adolescent well-being unaddressed.

### Common progress and enabling factors

The experiences of Bulgaria, Colombia, Malawi, and Nigeria reflect both the potential and the persistent challenges in translating global commitments into national action for adolescent well-being. While each country's context is unique, our analysis reveals shared patterns of progress and common barriers, offering valuable insights into implementation dynamics.

A major takeaway is the importance of policy formalization and political intent. All four countries demonstrated a clear commitment to prioritizing adolescents through new or updated national strategies and legal frameworks. For instance, Bulgaria’s National Youth Strategy (2021–30) (Republic of Bulgaria 2021) and Colombia’s Law 2328 (ICBF 2023b) provided robust legal foundations. Similarly, Malawi's increased budget for Reproductive, Maternal, Newborn, Child, and Adolescent Health and Nutrition (RMNCAH) (UNICEF Malawi 2024a, 2024b) and Nigeria's National Youth Policy signal strong political backing (Federal Ministry of Youth and Sports Development Nigeria 2019). This aligns with existing evidence that political will and sustained government commitment are critical precursors for long-term policy implementation (Jones et al. 2022). Our study adds a new dimension by showing that in some cases, political instability can significantly undermine even the most ambitious commitments by disrupting governance, fragmenting policy continuity, and weakening institutional capacity. This reflects broader research indicating that while political will is necessary, it is not sufficient; stability and governance quality critically influence policy outcomes in youth development. Our findings stress the need for greater attention to not just awareness of political contexts, but a strategic focus on governance quality when designing and implementing adolescent well-being policies, underlining the importance of creating mechanisms that can insulate youth policy from being easily reversed or defunded when the political landscape changes.

Furthermore, implementation is increasingly influenced by regional blocs that provide a critical layer of accountability. For Bulgaria, progress is nested within the European Union's Youth Strategy, while Nigeria and Malawi leverage the African Union's Agenda 2063 and its emphasis on the demographic dividend. These regional bodies provide a “scaffolding” for national action, offering extra-national monitoring and peer-learning opportunities that reinforce the sustainability of national pledges.

A second crucial insight is the growing recognition of the importance of youth participation and agency. All four countries have moved beyond simply consulting young people to genuinely co-creating policies and programs with them. For example, in Bulgaria, the UPSHIFT innovation program empowered adolescents to lead solutions to local problems (UNICEF 2025a), while Colombia’s participatory policymaking has directly shaped health services (PoD Consortium 2025). Similarly, Malawi's CAAP initiative (Amref Health Africa and PMNCH 2025) and Nigeria's youth-led accountability platforms (The Youth Pact 2023) show a clear shift toward seeing young people as active stakeholders in policy and program design and monitoring. This finding emphasizes the importance of legitimacy and responsiveness in public health interventions, which has increasingly highlighted the value of sustainable, meaningful youth participation beyond tokenistic consultation. It also demonstrates promising new “ways of working” where youth are not just recipients of services but co-leaders in their provision, actively shaping the development, implementation, and evaluation of programs that affect their lives. This co-leadership fosters empowerment, strengthens accountability, and bridges research-to-practice gaps in ways not fully captured in common, widespread practices that treat adolescents and youth as objects of intervention rather than agents of change.

### Common challenges and persistent barriers

Despite strong commitments, a gap persists between policy intent and action. Implementation is hindered by fragmented services and weak mechanisms for cross-sectoral coordination; Bulgaria's disjointed social systems and Malawi's institutional inertia illustrate these challenges (Stoyanov and Nakov 2023, Global Health Research Group 2025). Chronic underinvestment and reliance on donor funding further limit scale and sustainability, as seen in Malawi (Muula 2025), highlighting that financial commitment must match policy ambition. Socio-economic disparities continue to constrain access for vulnerable adolescents, particularly in rural, marginalized, and ethnic minority communities in Colombia and Malawi (Hara 2018, UNICEF 2023). Data gaps and weak accountability mechanisms hinder monitoring and feedback, while political volatility disrupts continuity and long-term planning, underscoring the need for legally binding, institutionally embedded frameworks.

Tangible progress emerges when political will, financing, and accountability converge. Examples from Malawi's increased health allocations and Nigeria's youth-led advocacy demonstrate how countries can translate global pledges into measurable national impact. Our findings underscore persistent structural barriers such as fiscal dependency, governance fragmentation, and intergovernmental coordination challenges that require to be addressed; and the critical need for tailored strategies to strengthen institutional coherence and sustainable financing for adolescent well-being programs.

### Navigating the global landscape: risks and backlash

Despite these gains, the current global landscape poses significant threats to implementation. The rise of fiscal austerity and international funding cuts (e.g. significant reductions in Official Development Assistance) directly endangers the sustainability of multi-sectoral programs in countries like Malawi and Nigeria, which rely heavily on external support.

Furthermore, a growing anti-rights backlash in several regions threatens to undermine progress in sensitive domains such as SRHR and gender equality. Without legally binding, institutionally embedded frameworks and diversified financing, adolescent commitments remain vulnerable to these shifting political and financial tides. This underscores the need for strategic focus on governance quality to create mechanisms that can insulate youth policy from being easily reversed when the political landscape changes.

### Recommendations for policy and practice

To bridge the gap between commitment and implementation and impact, and to accelerate progress toward comprehensive adolescent well-being, stakeholders should prioritize the following actionable recommendations:

#### Strengthen and diversify financial investment

Governments and international partners must move beyond initial pledges to ensure sustained, predictable, and targeted financial flows for adolescent well-being across all five domains of the adolescent well-being framework (PMNCH, UNFPA, UNICEF and WHO 2024). This includes increasing domestic budgetary allocations, ensuring that programs to promote adolescent well-being are not over-reliant on one or a small number of external funders, exploring innovative financing mechanisms (e.g. earmarking a share of lottery revenues, offering tax incentives for donations to accredited youth-led organizations, etc.), and directing funds to address comprehensive initiatives that span adolescent and youth health, education, social protection and empowerment. Investment in the well-being of adolescents must be seen as a long-term strategic imperative, not a discretionary expenditure.

#### Institutionalize multi-sectoral governance and coordination

Establish and empower formal inter-ministerial bodies and multi-stakeholder platforms with clear mandates, resources, and accountability frameworks to drive integrated policy, planning and implementation (WHO et al. 2025a, 2025b). This requires developing common indicators, standardized data-sharing protocols, and fostering multi-sectoral approaches (George et al. 2021) that embed adolescent well-being considerations across all relevant government sectors. Beyond formal structures, a culture of trust and shared vision must be cultivated among diverse actors to facilitate genuine collaboration, drawing lessons from successful multi-stakeholder platforms, such as those that mandate inclusive country-level coordination and cross-sectoral alignment (*see recommendation* *‘Prioritize and resource meaningful youth engagement and accountability’*).

#### Prioritize and resource meaningful youth engagement and accountability

Systematically integrate adolescents and youth as co-creators, implementers, and monitors of policies and programs that affect them (FP2020, IYAFP and PMNCH 2018). This involves providing meaningful platforms for participation (e.g. youth representation on national, regional, and local decision-making bodies, establishing and supporting youth councils and citizens' assemblies including youth), investing in youth-led organizations, and strengthening their capacity for advocacy and data-driven accountability (e.g. supporting the development and use of citizens' accountability dashboards). Governments should establish transparent mechanisms for reporting on commitments, with adolescents and youth actively involved in monitoring progress and being encouraged and enabled to hold stakeholders accountable.

#### Enhance data systems for disaggregated measurement and learning

Countries should invest in robust national data collection systems capable of producing age- and sex- and gender-disaggregated data, with specific indicators tailored to adolescent well-being across all domains (WHO et al. 2025a, 2025b). These data are crucial for evidence-driven policymaking, identifying disparities, and tracking progress. Furthermore, a learning culture should be fostered that uses evaluation findings to adapt and refine interventions, recognizing that policy translation is an iterative process requiring continuous adjustment.

#### Address underlying structural factors that undermine adolescent well-being

Countries should recognize that adolescent well-being is deeply intertwined with broad social, economic, and political determinants (WHO et al. 2025b). Policy responses must therefore adopt a systems-thinking approach, addressing root causes such as poverty, conflict, gender inequality, and weak educational systems. This requires integrated and adaptive policy responses that acknowledge and proactively manage the complex synergies and trade-offs inherent in multi-sectoral policy execution (Kuruvilla et al. 2018), ensuring that progress in one area does not inadvertently undermine others.

#### Mitigate the impact of political instability

Countries need to develop and safeguard long-term strategic frameworks for adolescent well-being that transcend political cycles, ensuring continuity and sustainability of reforms regardless of government changes (Baird et al. 2024). Developing cross-party consensus on fundamental child and adolescent rights and well-being policies should be prioritized to insulate them from political shifts. Efforts should also be made to strengthen the capacity of civil society organizations and international partners to provide consistent support and advocacy for adolescent well-being, acting as a stabilizing force amidst political fluctuations.

#### Conduct regular accountability assessments

To ensure that commitments are not merely aspirational pledges, studies similar to this one should be conducted in the future, repeated at regular intervals (e.g. every 2–3 years). These assessments should measure progress against each country’s specific commitments, using a standardized methodology to provide both quantitative and qualitative data. The findings should be used not only to hold stakeholders accountable but also to provide constructive suggestions for improvement and to celebrate progress where it has been made, thereby sustaining momentum and encouraging further action.

### Strengths and limitations

A key strength of this study is its multi-method qualitative approach, which combines a review of national commitments with in-depth qualitative case studies. We integrated descriptive quantitative mapping of 24 countries with in-depth qualitative case studies. This approach moves beyond simply tallying pledges to explore the “why” and “how” of early implementation across diverse country contexts. Triangulating official documents with real-world narratives allowed us to capture both systemic patterns and the lived experiences of implementation. The selection of Bulgaria, Colombia, Malawi, and Nigeria provides geographical and developmental diversity, enhancing the relevance and transferability of our findings. The involvement of KIs as co-authors further strengthens the internal validity and credibility of the findings.

However, the early-stage nature of this analysis conducted less than 2 years after commitments were made, means our assessment of progress is preliminary, and full policy impacts may take many years to emerge. Focusing on only four countries provides depth but limits generalizability, as each nation's political, social, and economic realities are unique. Challenges in obtaining comprehensive, disaggregated data on adolescent well-being, as well as potential reporting biases in government documents and public statements, further constrain the analysis. While this study offers valuable insights into translating commitments into action, further research is needed to track long-term impact and implementation dynamics across a broader range of countries.

## Conclusion

The 2023 GFA marked a pivotal moment, successfully catalyzing unprecedented, explicit commitments from a diverse array of stakeholders to prioritize adolescent well-being (PMNCH 2023c, 2023d). This global recognition, underpinned by the UN H6+ definition and comprehensive adolescent well-being framework (Ross et al. 2020) and the youth-driven Agenda for Action for Adolescents (PMNCH 2023a), represents a significant maturation of global health policy, moving beyond fragmented interventions to a holistic and integrated understanding of adolescent needs. However, the analysis of early national efforts in Bulgaria, Colombia, Malawi, and Nigeria reveals that, while concrete steps toward implementing these commitments have been taken in all four countries, the effective translation of these global pledges into sustainable local action is a complex, non-linear process fraught with systemic challenges. Tangible progress emerges only when political will, targeted financing, and youth-led accountability converge. To ensure the momentum of the GFA is not lost, regular accountability assessments (every 2–3 years) should be conducted to track the long-term institutionalization of these commitments. Future research should aim to extend this evaluative lens to a broader range of the GFA-participating nations, providing the longitudinal evidence needed to track the institutionalization of adolescent well-being across diverse political and economic landscapes.

By embracing the recommendations from our paper, stakeholders can help shape a more resilient and prosperous future for the largest generation of young people in history (Baird et al. 2025), by collectively working toward translating global aspirations into tangible, equitable, and sustainable improvements in adolescent well-being.

## Reflexivity statement

This study was conducted by a multi-disciplinary team with expertise in global health policy, adolescent health, social sciences, and advocacy. We acknowledge that our institutional affiliations and prior involvement in adolescent health initiatives shaped the study's framing and interpretation of findings. To mitigate bias, we drew on diverse team perspectives, triangulated data sources, and engaged country-level stakeholders to validate interpretations. Our goal has been to present evidence-informed findings that reflect diverse contexts and center the voices of adolescents and youth.

## Data Availability

The data underlying this article include publicly available policy documents, strategic plans, and official commitment records from the 2023 Global Forum for Adolescents, which can be accessed via the PMNCH website. Qualitative data derived from structured key informant interviews are not publicly available to protect the privacy and confidentiality of the participants.
